# Single-cell qPCR demonstrates that Repsox treatment changes cell fate from endoderm to neuroectoderm and disrupts epithelial-mesenchymal transition

**DOI:** 10.1371/journal.pone.0223724

**Published:** 2019-10-10

**Authors:** Qiuhong Li, Qingsong Huang

**Affiliations:** 1 School of Biosciences and Biopharmaceutics, Guangdong Pharmaceutical University, Guangzhou, China; 2 South China Institute of Stem Cell Biology and Regenerative Medicine, Guangzhou Institutes of Biomedicine and Health, Chinese Academy of Sciences, Guangzhou, China; Western University, CANADA

## Abstract

A definitive endodermal cell lineage is a prerequisite for the efficient generation of mature endoderm derivatives that give rise to organs, such as the pancreas and liver. We previously reported that the induction of mesenchymal definitive endoderm cells depends on autocrine TGF-β signaling and that pharmacological blockage of TGF-β signaling by Repsox disrupts endoderm specification. The definitive endoderm arises from a primitive streak, which depends largely on TGF-β signaling. If the TGF-β pathway is blocked by Repsox, cell fate after the primitive streak induction is so-far unknown. We report here, that an induced primitive streak cell-population contained many T/SOX2 co-expressing cells, and subsequent inhibition of TGF-β signaling by Repsox promoted neuroectodermal cell fate, which was characterized using single-cell qPCR analysis and immunostaining. The process of epithelial-to-mesenchymal transition, which is inherent to the process of definitive endoderm differentiation, was also disrupted upon Repsox treatment. Our findings may provide a new approach to produce neural progenitors.

## Introduction

Differentiation of human pluripotent stem cells (hPSCs) into definitive endoderm (DE) is the critical first step for generating visceral organs, such as liver, pancreas, gut, and lungs [1]. Most protocols for efficient production of DE cells employ exogenous Wnt and recombinant activin A to induce a primitive streak (PS) intermediate within 24 h, followed by continued TGF-β/activin/nodal signaling for the subsequent 2–5 days. By systematically optimizing the differentiation protocol, Loh et al. were able to differentiate hPSCs into > 98% pure SOX17-expressing DE cells within 48 h [2, 3]. In vertebrate embryos and during hPSC differentiation, activation of TGF-β/activin/nodal signaling by activin A is imperative for DE specification [4].

During vertebrate gastrulation, epiblast cells undergo an epithelial-to-mesenchymal transition (EMT) at the primitive streak. During the period of *in-vitro* endoderm differentiation, EMT also occurs with noticeable changes in cell morphology and upregulation of EMT-related genes [5]. We observed that endogenous TGF-β1 was largely secreted during endoderm specification, and pharmacological inhibition of TGF-β/activin/nodal signaling disturbed DE formation and EMT events.[6]

Pluripotent epiblast cells can give rise to three germ layers (ectoderm, mesoderm, and endoderm), and neural tissues are traditionally considered to mainly originate from the ectoderm. The discovery of a bipotent neuro-mesodermal progenitor (NMp), which is considered to occur within the primitive streak-associated epiblast and is bipotential for the posterior neural plate and the paraxial mesoderm, however, challenges the traditional notion [7, 8]. NMps, also referred to as axial stem cells, are thought to co-express the neural progenitor marker SOX2 and the early mesodermal marker brachyury (T) in the embryo [9]. Axial stem cells can give rise to neural lineages by persistent activation of SOX2 [10]. It is interesting that successful NMps can be induced from mouse epiblast stem cells (EpiSCs) when cultured in the presence of activin [11]. However, it remains unknown whether co-expressing T and SOX2 cells from hPSCs can be generated following PS induction by activin; moreover, cell fate changes due to TGF-β inhibition caused by Repsox after PS induction are not comprehensively understood.

Here, we report that numerous cells co-expressing T and SOX2 were observed following PS induction, and the subsequent efficient inhibition of TGF-β/activin/nodal signaling by Repsox promoted neuroectoderm formation, which can give rise to neural rosettes. Most DE-specific markers were not up-regulated in the presence of Repsox, and EMT events were also scarce. Based on these findings, we propose a model explaining the mechanism underlying the effects of Repsox.

## Materials and methods

### Cell culture and differentiation

Undifferentiated human H1 embryonic stem cells (WiCell) were routinely cultured on Matrigel (BD Biosciences, San Jose, USA; cat. no. 354277) in mTeSR1 medium (STEMCELL Technologies Vancouver, Canada; cat. no. 05850). Cultures were manually passaged from 1:6 to 1:12 using Accutase (Sigma, St. Louis, USA; cat. no. A6964) every 4–7 days. Monolayer, feeder-free definitive endoderm differentiation was conducted for three days in RPMI 1640/B27 minus insulin medium (Thermofisher Scientific, Massachusetts, USA; cat. no. 11875093 and cat. no. A18956-01) supplemented with 100 ng/mL activin A (Peprotech, Rocky Hill, USA; cat. no. A120-14E) as described previously [6]. After PS induction (day 0–1), cells were treated with 2 μM Repsox (Sigma; cat. no. R0158) for two days; Repsox selectively inhibits the TGF-β type I receptor/ALK5.

For further neural differentiation [12, 13], cultures were treated using N2B27 differentiation medium (1:1 of DMEM/F12 supplemented with 1% N2 [Thermofisher Scientific; cat. no. 17502048] and neurobasal medium [Thermofisher Scientific; cat. no. A24775-01] supplemented with 2% B27 [Thermofisher Scientific; cat. no. 17504044]) in the presence of 5 μM SB431542 (Selleck Chemicals, Houston, USA; cat. no. S1067), 1 μM Dorsomophin (Selleck Chemicals; cat. no. S7306) and 5 μg/ml human insulin (Sigma; cat. no. I9278) for eight days. Cells were then split and cultured in N2B27 differentiation medium without SB431542 and Dorsomophin until neural rosettes were observed, and 50 ng/ml bFGF (Gibco; cat. no. 13256029) was added to improve the growth of neural rosettes. Neural rosettes were then enriched to form neurospheres, which were cultured in N2B27 medium containing 20 ng/ml bFGF and 20 ng/ml EGF (Peprotech; cat. no. AF-100-15). For further neural differentiation, the passaged neurosperes were dissociated and plated on Matrigel-coated coverslips. Cells were then cultured in N2B27 medium with 10 ng/ml BDNF (Peprotech; cat. no. 450–02), 10 ng/ml GDNF (Peprotech; cat. no. 450–01), 10 ng/ml CNTF (Peprotech; cat. no. 450–13), 10 ng/ml IGF1 (Peprotech; cat. no. AF-100-11) and 1μM cAMP (Sigma; cat. no. D-0260) for 2 weeks.

### Immunostaining

Cells were fixed at 18–25°C in 4% wt/vol paraformaldehyde in phosphate-buffered saline (PBS) for 30 min; then, they were washed, blocked, and permeabilized using a blocking solution (PBS containing 10% fetal bovine serum and 0.3% Triton X-100) for 30 min. Primary and secondary antibodies were diluted in the blocking solution and were incubated at 4°C overnight and at room temperature for 1 h, respectively. Cells were washed twice and stained with DAPI (Sigma; cat. no. D9542) for 5 min, and then for photographing using Zeiss LSM 710 confocal microscope (Carl Zeiss). The following antibodies and dilutions were used: goat anti-SOX17, 1:400 (R&D systems, Minneapolis, USA; cat. no. AF1924); rabbit anti-E-cadherin, 1:100 (ABclonal Biotechnology, Wuhan, China; cat. no. A0965); mouse anti-N-cadherin, 1:100 (BD Bioscience; cat. no. 610920); rabbit anti-brachyury, 1:100 (Cell Signaling Technology, Massachusetts, USA; cat. no. 81694); goat anti-brachyury, 1:100 (R&D systems; cat. no. AF2085); rabbit anti-SOX2, 1:100 (Cell Signaling Technology; cat. no. 34516); rabbit anti-PAX6, 1:100 (Sigma; cat. no. HPA030775); rabbit anti-TUJ1, 1:1000 (Convance; cat. no. PRB-435P); mouse anti-MAP2, 1:1000 (Millipore; cat. no. MAB3418); rabbit anti-GFAP, 1:1000 (Sigma; cat. no. SAB4501162); Alexa Fluor® 488 donkey anti-goat IgG, 1:400 (Thermofisher Scientific; cat. no. A11055); Alexa Fluor® 568 donkey anti-rabbit IgG, 1:400 (Thermofisher Scientific; cat. no. A10042); Alexa Fluor® 568 donkey anti-mouse IgG, 1:400 (Thermofisher Scientific; cat. no. A10037).

### Flow cytometry

Cells were separated individually using Accutase for 5–10 min and were re-suspended in 2% fetal bovine serum (buffer) to prevent unspecific antibody binding. Cells were labeled using CD325 (Thermofisher Scientific; cat. no. 17-3259-42) for 30 min on ice. Single cells were then fixed in 4% paraformaldehyde for 10 min and permeabilized in 0.1% wt/vol Saponin (Sigma; cat. no. S4521) in PBS for 45 min. Cells were then incubated with SOX17 (BD Biosciences; cat. no. 562205) in a FACS buffer (2% fetal bovine serum in PBS) for 30 min on ice. Control samples were stained using isotype-matched control antibodies. To detect the expression of T in activin-induced PS cultures, the fixed and permeabilized cells were incubated with human/mouse brachyury Alexa Fluor® 488-conjugated antibodies (R&D Systems; cat. no., IC2085G) for 1 h at 37°C. All cells were analyzed using an Accuri C6 device (BD Biosciences).

### Quantitative reverse-transcription polymerase chain reaction (RT-qPCR)

Total RNA was isolated from samples in triplicate using TRIzol (Thermofisher Scientific; cat. no. 15596026), and 2 μg RNA was used for reverse-transcription with ReverTrace (TOYOBO, Osaka, Japan; cat. no. 34520B1). The produced cDNA was diluted for use as PCR template, and PCR reactions were performed using a SYBR® Premix Ex Taq^™^ Kit (Takara Bio, Kusatsu, Japan; cat. no. RR420A) and a CFX96 Touch^™^ Real-Time PCR Detection System (Bio-Rad). Gene expression levels were normalized against the expression level of *GAPDH*. Primer sequences are shown in the supplementary file S1 Data.

### Enzyme-linked immunosorbent assay (ELISA)

The concentration of TGF-β1 in the cell culture medium was determined using an ELISA kit (R&D Systems; cat. no. DB100B). Cell culture medium supernatant was assayed following the manufacturer’s instructions. Optical density was measured at 450 nm using a microplate reader (Mithras2 LB943, Berthold Technologies), and chemokine concentrations were quantified using MikroWin 2010 software (version 5.14, Labsis Laborsysteme GmbH).

### Cell migration assay

An artificial wound area in the differentiated and mostly confluent cell monolayer was created using a 200 μL pipet tip. The cultures were then rinsed twice using DMEM/F12 and were subsequently covered with fresh RPMI/B27-minus and activin A medium with or without Repsox. At 0 and 24 h, cells were assessed and photographed under an inverted phase contrast microscope (OLYMPUS IX51, Olympus Optical Co., Ltd).

### Single cell qPCR

Cells treated with Repsox were captured using the C1 System (Fluidigm) and subsequently lysed; cDNA was synthesized and pre-amplification and gene expression were assessed using the Ambion Single Cell-to-CT Kit (Thermofisher Scientific; cat. no. PN 4458237), C1 Single-Cell Auto Prep Module 2 Kit (Fluidigm, San Francisco, USA; cat. no. PN100-5519), TaqMan® Gene Expression Master Mix (Thermofisher Scientific; cat. no. 4369016), and TaqMan® Gene Expression assays (20X, Thermofisher Scientific), respectively. Inventoried TaqMan primers were used, and data was analyzed as described previously [6].

### Statistical analyses

Experiments were performed using three biological repeats, whenever possible. Data are shown as mean ± standard deviation (s.d.). The effects of treatments were tested using unpaired two-tailed Student’s t-tests. Statistical significance is reported as P < 0.05.

## Results

**Fig 1:**
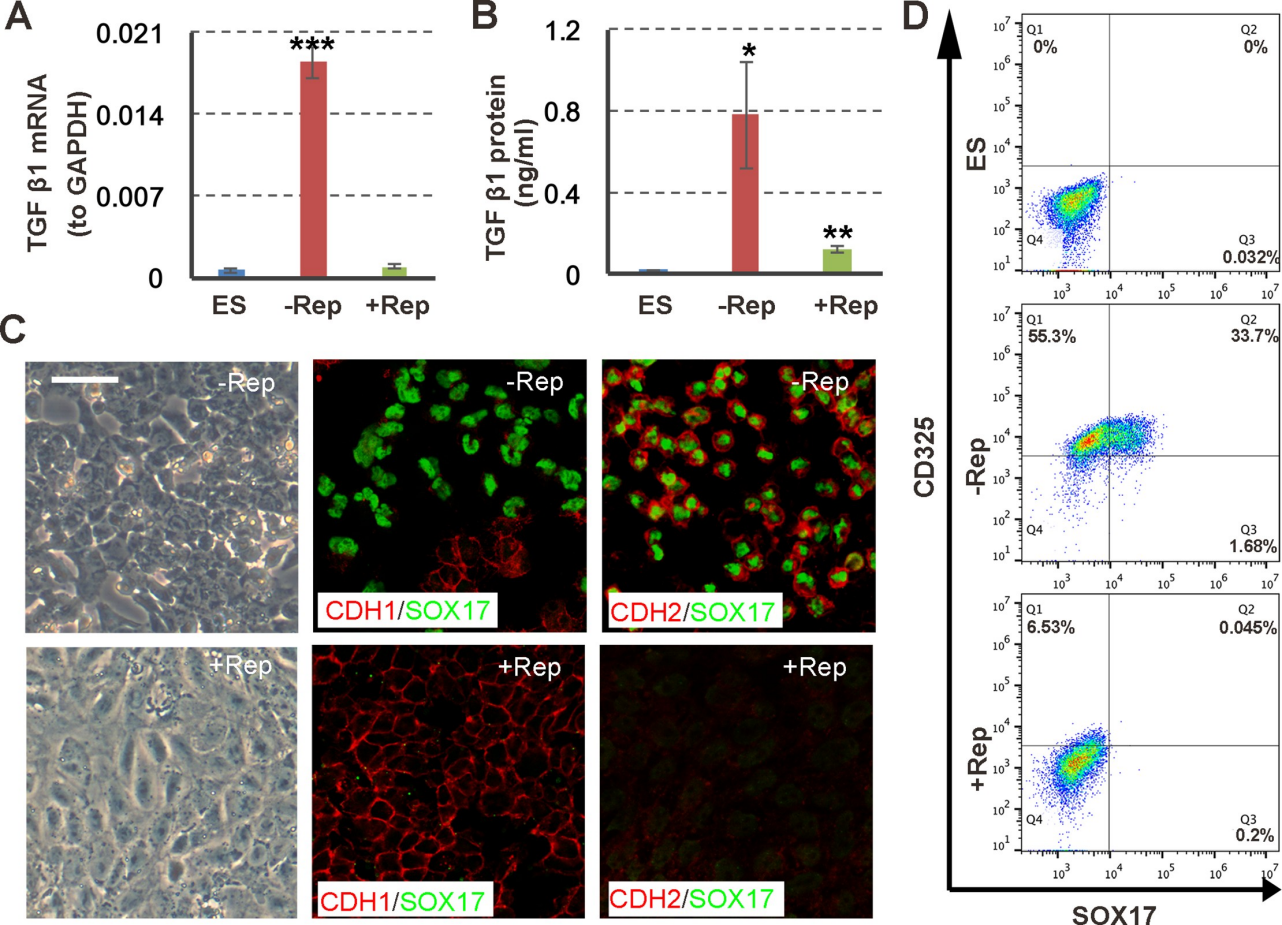
Inhibition of TGF-β signaling after PS induction disrupts endoderm specification. (A) *TGF-β1* expression levels in un-induced human embryonic stem cells (hESCs) assessed by RT-qPCR, or in activin differentiation medium with or without Repsox (Rep) treatment after PS induction (day 0–1). The expression level of *GAPDH* was arbitrary set as 1. Shown are the means ± s.d. of three replicates. *** P < 0.001, compared to ES. (B) Concentrations of secreted TGF-β1 in medium containing activin A with or without Repsox (Rep) on day 3, measured using an ELISA. Shown are the means ± s.d. of three replicates. * P < 0.05; ** P < 0.01, compared to ES. (C) Photos and immunofluorescence images of activin A-induced cells with or without Repsox (Rep) treatment. The scale bar indicates 20 μm. (D) Flow cytometry analysis of activin A-induced cells with or without Repsox treatment.

DE cells were produced at up to 80% confluency of the cultures in the presence of activin A, and an apparent EMT was observed. We detected a strong increase in *TGF-β1* gene expression and a high level of TGF-β1 protein secretion into the medium after activin A induction. The addition of Repsox after PS induction led to a sharp decrease in *TGF-β1* gene expression (Fig 1A) and TGF-β1 concentrations (Fig 1B). The cells stained for immunofluorescence (IF) analysis showed a loss of the DE marker SOX17 and of the EMT-related protein N-cadherin (CDH2) in the presence of Repsox; however, the epithelial marker E-cadherin (CDH1) was maintained consistently. CDH1 and SOX17 expression levels appeared to be mutually exclusive (Fig 1C). The flow cytometry results indicated that about 33.7% of the SOX17+CD325+ cells were produced after activin A induction, and SOX17+CD325- cells were scarce, suggesting a mesenchymal status of the DE cells (Fig 1D). In the Repsox treatment, no SOX17 was detected, and CD325 was substantially downregulated, which was in line with the IF results.

**Fig 2:**
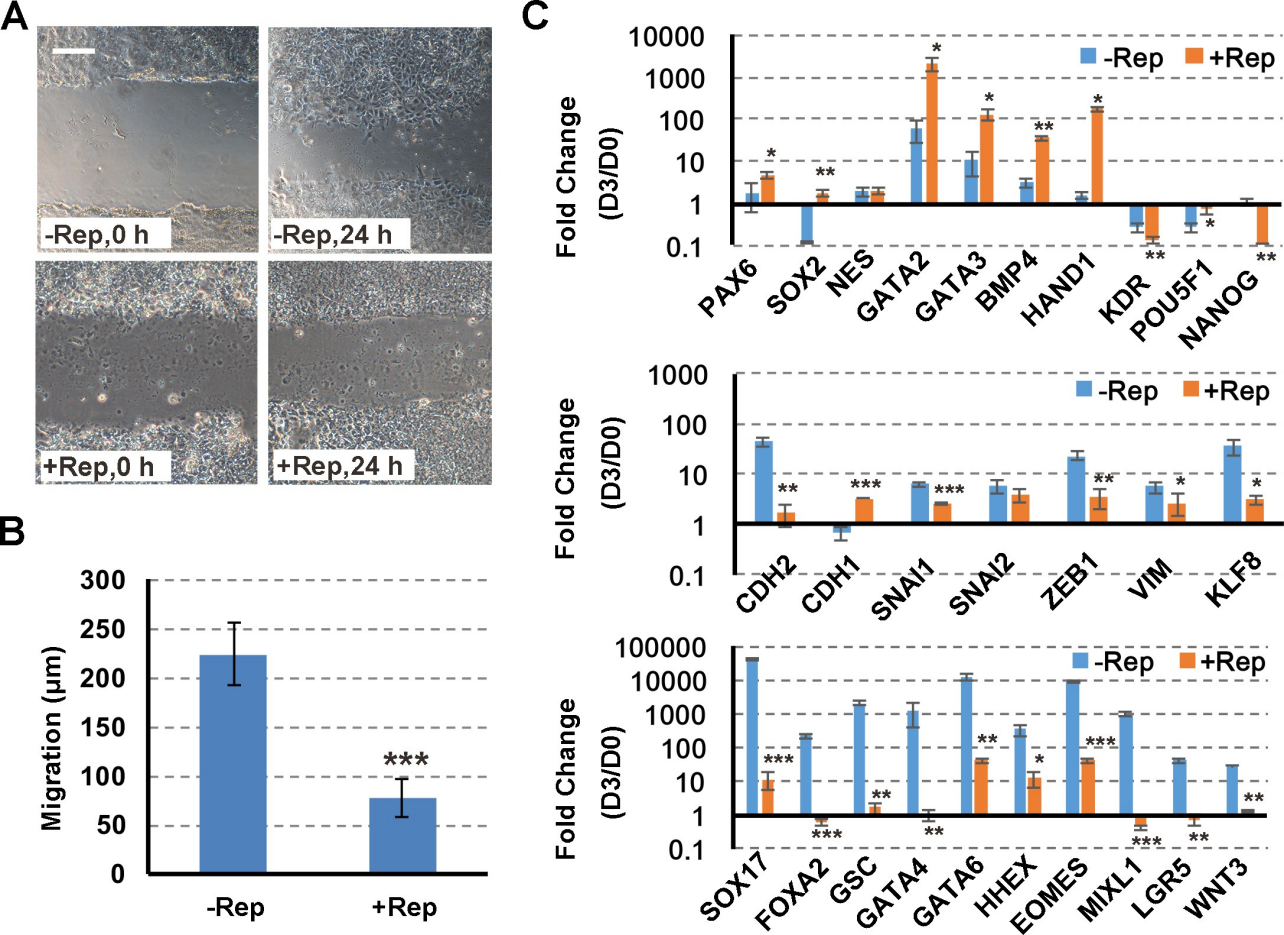
Inhibition of TGF-β signaling after PS induction blocks the EMT but promotes ectoderm/mesoderm cell fates. (A, B) Scratch assays for activin A-induced cells with or without Repsox (Rep) treatment. The scale bar indicates 100 μm. Shown, are the means ± s.d. of three technical replicates. *** P < 0.001, compared to the control. (C) RT-qPCR analysis for the indicated genes with or without Repsox (Rep) treatment. Shown are the means ± s.d. of three replicates. * P < 0.05; ** P < 0.01; *** P < 0.001, compared to the control.

A scratch assay (Fig 2A and 2B) was used to investigate the effects of Repsox on cell migration activity. Cells showed very limited migration activity (77 ± 20 μm/24 h) in the Repsox treatment population, indicating their loss of the mesenchymal phenotype. We investigated the inhibition effect of Repsox on endoderm induction using a qPCR (Fig 2C). The expression of the neuroectoderm marker genes *PAX6* and *SOX2* was significantly increased in the Repsox treatment after PS induction, as were the mesoderm-related markers *GATA2/3*, *HAND1*, and *BMP4* [9, 14, 15]. In addition, *NANOG* was downregulated compared to hESCs (day 0). The mesenchymal markers *CDH2*, *SNAI1*, *ZEB1*, and *VIM* were also blocked by Repsox. The early and late endoderm markers *SOX17*, *FOXA2*, *GATA4*, and *GATA6* were not substantially up-regulated after Repsox treatment, nor were the mesoendoderm markers *HHEX*, *EOMES*, *MIXL1*, and *WNT3*.

**Fig 3:**
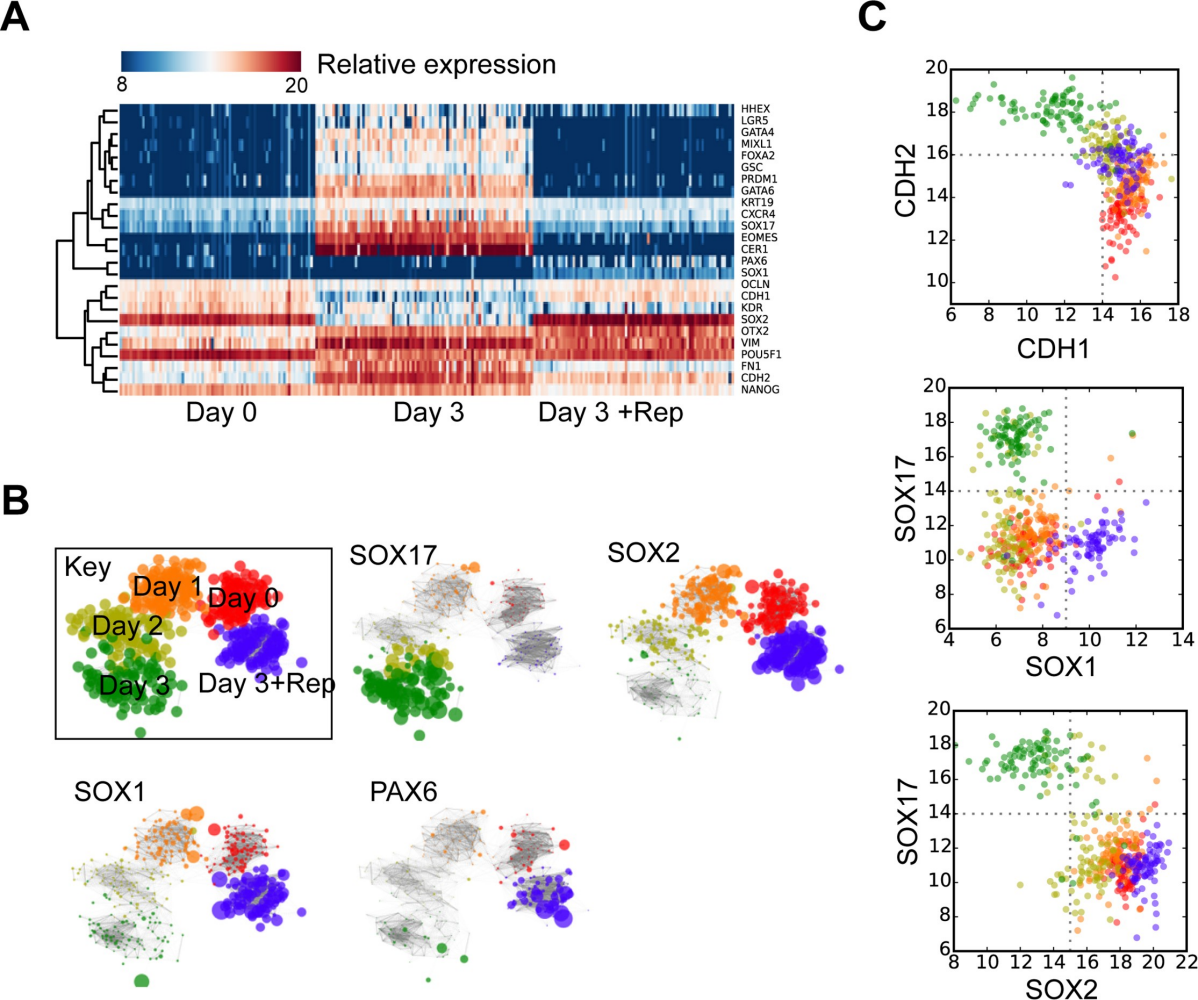
Single cell qPCR on cells treated with activin A and Repsox (Rep) after PS induction, in combination with previously published data. (A) Expression of selected genes. (B) Relation networks of hESCs (day 0), DE differentiation (day 3), and DE induction with Repsox for two days (treatment on day 3). Expression of *PAX6*, *SOX1*, *SOX2*, and *SOX17* is indicated, respectively. Each cell is a node and node sizes are 2^[relative expression]^. (C) Expression levels of *CDH1* versus *CDH2*, *SOX1* versus *SOX17*, and *SOX2* versus *SOX17*. Colors are the same as used in panel B.

The gene expression tests revealed that epithelial–mesenchymal–epithelial transition and acquisition of endoderm markers can be simultaneously disrupted by Repsox, an TGF-βR1 (ALK5) inhibitor, and differentiated cells seemed to express ectoderm and mesoderm markers instead. In order to characterize these cells, we performed single-cell qPCR on 81 cells to measure gene expression in individual cells treated with activin A and Repsox after PS induction. For data analysis, we used previously published single-cell qPCR data to produce relational networks of gene expression of those cells [6]. The cell population treated with Repsox was synchronized and homogeneous (Fig 3A). The expression of pluripotency gene *NANOG* in cell population treated with Repsox was obviously downregulated compared to hESCs. TGF-β signaling pathway inhibitor significantly blocked the acquisition of endoderm marker genes, particularly that of *SOX17* (Fig 3B). After the Repsox treatment, the ectoderm marker genes *PAX6* and *SOX1* were up-regulated, as was *SOX2*, which was also up-regulated compared to hESCs (day 0; Fig 3B). The variation in gene expression of the hematopoiesis-related marker *GATA2* [16] and of the mesoderm markers *HAND1* [3, 17, 18] and *HAND2* [16] was scarcely observed in single cells (S2 Data). For visualization, we produced scatter plots of individual cell expression of *CDH1* against *CDH2*, *SOX1*, and of *SOX2* against the DE marker gene *SOX17* (Fig 3C). We showed that increasing ectoderm marker levels were associated with decreasing endoderm marker levels upon the addition of Repsox after PS induction, as *SOX1* and *SOX2* expression and *SOX17* expression were mutually exclusive, and no EMT was observed as measured by the ratio of *CDH1* and *CDH2* expression.

**Fig 4:**
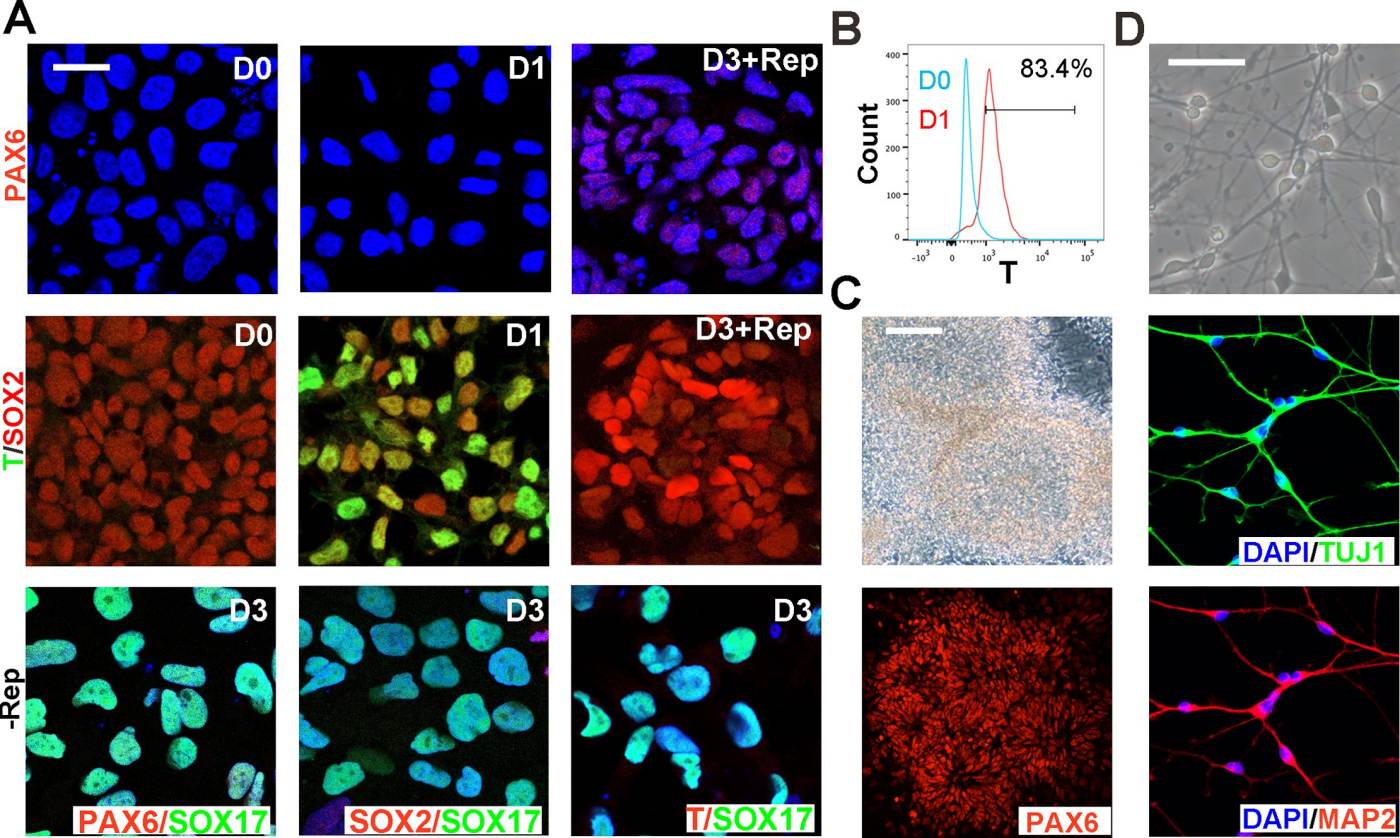
Effects of a Repsox treatment on protein expression and functional characterization of the early neuroectoderm. **(A)** Immunofluorescence images of activin-induced cells with or without Repsox (Rep) treatment on day 0, 1, and 3, respectively. The scale bar indicates 20 μm. **(B)** Flow cytometry analysis of T expression in activin A-induced PS cells. **(C)** Photos and immunofluorescence images of the neural rosette derived from induced neuroectoderm. The scale bar indicates 100 μm. **(D)** Photos and immunofluorescence images of neural cells derived from neurospheres. The scale bar indicates 100 μm.

Both RT-qPCR and single-cell qPCR analysis revealed that TGF-β/activin/nodal signaling inhibition by Repsox after PS induction resulted in an upregulation of neuroepithelial markers such as *SOX2*, *PAX6*, and *SOX1*. The immunostaining results showed that many cells treated with Repsox expressed the early neuroectoderm markers PAX6 and SOX2 on day 3 (Fig 4A), which was scarcely detected in the DE population. After the primitive streak induction, numerous cells were observed to co-express SOX2 and T (83.4%; Fig 4A and 4B); however, T expression decreased rapidly on day 3 with and without the Repsox treatment. In order to further characterize the obtained early neuroectoderm cells, we continued culturing them in N2B27 basal medium with SB431542 and Dorsomorphin for eight days, and insulin was also added to improve the induction process [19]. Cells were then split and cultured in N2B27 basal medium with bFGF until neural rosettes were observed[19, 20]. We observed the expression of the neuroepithelial marker PAX6 in the neural rosettes by immunostaining (Fig 4C). Neurospheres derived from these rosettes were further differentiated to neural cells, which were both TUJ1-positive and MAP2-positive (Fig 4D). The astrocyte marker GFAP can hardly be detected, since the neuroepithelium mainly gives rise to neurons upon differentiation and gliogenesis may be achieved after a long-time culture[21]. Based on the results above, the early neuroectoderm can be generated after a Repsox treatment following PS induction.

## Discussion

Efforts have been made to efficiently differentiate hPSCs into DE cells [2, 5, 22–24]. For nearly all of the methods aiming to do this, TGF-β/activin/nodal signaling activated by activin is essential for DE specification; however, it is insufficient for producing homogeneous DE cells even combined with the use of additional growth factors [25]. Single-cell qPCR is a powerful method that can be used to assess specific gene expression at single-cell resolution and reveal cellular heterogeneity, and this method has been widely applied in the biomedical field [26–28].

DEs were reported to arise from PS *in vivo*, as well as NMps [8, 29]. Tsakiridis et al. reported the generation of NMp-like cell populations from mouse EpiSCs cultured in activin and FGF2 *in vitro* [11]. In our study, numerous T/SOX2-positive cells were detected in the activin A-induced primitive streak, the formation of which depended on endogenous Wnt with T as a specific marker [2]. We thus hypothesized that these NMp-like cells may be biopotential for neural and mesoderm differentiation.

Recent studies demonstrated that NMps can subsequently be differentiated towards neural fate with RA and a sonic hedgehog agonist *in vitro*[30, 31]. Surprisingly, activin/nodal inhibition was found to efficiently promote neural conversion from hPSCs [32]. Based on these reports, we aimed to determine whether Repsox can steer T/SOX2-positive cells in an activin-induced PS to be neural progenitors.

We previously reported that activin-induced DE formation was associated with a synchronous EMT mediated by autocrine TGF-β signaling, and blockage of TGF-β/activin/nodal signaling by Repsox during three days of differentiation inhibited EMT and DE formation [6]. Here, we investigated cell fate changes accompanying pharmacological inhibition of TGF-β signaling in activin A-induced PS cultures, particularly at single-cell resolution. We found that inhibition of TGF-β/activin/nodal signaling by Repsox after PS induction promoted neuroectodermal cell fates with the loss of mesenchymal characteristics. Our method largely differs from the classical derivation of PAX6 neural progenitor cells by dual SMAD inhibition [33]. We thus propose a model explaining the mechanism underlying the effects of Repsox treatments. The cell population after PS induction contained stem cells co-expressing SOX2 and T, which can give rise to both neuroectoderm and mesoderm. Repsox may facilitate the generation of neuroectoderm by the persistent activation of SOX2, which by itself was demonstrated to generate induced neural stem cells from mouse and human fibroblasts [34].

Taken together, our results demonstrate that the pharmacological inhibition of TGF-β/activin/nodal signaling in activin A-induced PS cultures leads to substantial cell fate changes from DE cells to neuroectoderm lineages with the disappearance of noticeable EMT events accompanying DE formation.

## Supporting information

S1 Data Primers for RT-qPCR. (XLSX)

S2 Data Single-cell qPCR. (XLSX)

S3 Data Interactive plot data. (XLSX)

## References

[1] Yiangou L, Ross ADB, Goh KJ, Vallier L. Human Pluripotent Stem Cell-Derived Endoderm for Modeling Development and Clinical Applications. Cell Stem Cell. 2018;22(4):485–99. 10.1016/j.stem.2018.03.016 29625066

[2] Loh KM, Ang LT, Zhang J, Kumar V, Ang J, Auyeong JQ, et al Efficient endoderm induction from human pluripotent stem cells by logically directing signals controlling lineage bifurcations. Cell Stem Cell. 2014;14(2):237–52. 10.1016/j.stem.2013.12.007 24412311 PMC4045507

[3] Loh KM, Chen A, Koh PW, Deng TZ, Sinha R, Tsai JM, et al Mapping the Pairwise Choices Leading from Pluripotency to Human Bone, Heart, and Other Mesoderm Cell Types. Cell. 2016;166(2):451–67. 10.1016/j.cell.2016.06.011 27419872 PMC5474394

[4] Bogacheva MS, Khan S, Kanninen LK, Yliperttula M, Leung AW, Lou YR. Differences in definitive endoderm induction approaches using growth factors and small molecules. J Cell Physiol. 2018;233(4):3578–89. 10.1002/jcp.26214 29044512

[5] D'amour KA, Agulnick AD, Eliazer S, Kelly OG, Kroon E, Baetge EE. Efficient differentiation of human embryonic stem cells to definitive endoderm. Nat Biotechnol. 2005;23(12):1534–41. 10.1038/nbt1163 16258519

[6] Li Q, Hutchins AP, Chen Y, Li S, Shan Y, Liao B, et al A sequential EMT-MET mechanism drives the differentiation of human embryonic stem cells towards hepatocytes. Nat Commun. 2017;815166. 10.1038/ncomms15166 PMC5418622 28466868

[7] Tzouanacou E, Wegener A, Wymeersch FJ, Wilson V, Nicolas JF. Redefining the progression of lineage segregations during mammalian embryogenesis by clonal analysis. Dev Cell. 2009;17(3):365–76. 10.1016/j.devcel.2009.08.002 19758561

[8] Henrique D, Abranches E, Verrier L, Storey KG. Neuromesodermal progenitors and the making of the spinal cord. Development. 2015;142(17):2864–75. 10.1242/dev.119768 26329597 PMC4958456

[9] Pandolfi PP, Roth ME, Karis A, Leonard MW, Dzierzak E, Grosveld FG, et al Targeted disruption of the GATA3 gene causes severe abnormalities in the nervous system and in fetal liver haematopoiesis. Nat Genet. 1995;11(1):40–4. 10.1038/ng0995-40 7550312

[10] Takemoto T, Uchikawa M, Yoshida M, Bell DM, Lovell-Badge R, Papaioannou VE, et al Tbx6-dependent Sox2 regulation determines neural or mesodermal fate in axial stem cells. Nature. 2011;470(7334):394–8. 10.1038/nature09729 21331042 PMC3042233

[11] Tsakiridis A, Huang Y, Blin G, Skylaki S, Wymeersch F, Osorno R, et al Distinct Wnt-driven primitive streak-like populations reflect in vivo lineage precursors. Development. 2014;141(6):1209–21. 10.1242/dev.101014 24595287 PMC3943179

[12] Kim DS, Lee JS, Leem JW, Huh YJ, Kim JY, Kim HS, et al Robust enhancement of neural differentiation from human ES and iPS cells regardless of their innate difference in differentiation propensity. Stem Cell Rev. 2010;6(2):270–81. 10.1007/s12015-010-9138-1 20376579

[13] Liu J, Wang L, Su Z, Wu W, Cai X, Li D, et al A reciprocal antagonism between miR-376c and TGF-beta signaling regulates neural differentiation of human pluripotent stem cells. FASEB J. 2014;28(11):4642–56. 10.1096/fj.13-249342 25114173

[14] Tsai FY, Keller G, Kuo FC, Weiss M, Chen J, Rosenblatt M, et al An early haematopoietic defect in mice lacking the transcription factor GATA-2. Nature. 1994;371(6494):221–6. 10.1038/371221a0 8078582

[15] Goldman DC, Bailey AS, Pfaffle DL, Al Masri A, Christian JL, Fleming WH. BMP4 regulates the hematopoietic stem cell niche. Blood. 2009;114(20):4393–401. 10.1182/blood-2009-02-206433 19759357 PMC2777124

[16] Gifford CA, Ziller MJ, Gu H, Trapnell C, Donaghey J, Tsankov A, et al Transcriptional and epigenetic dynamics during specification of human embryonic stem cells. Cell. 2013;153(5):1149–63. 10.1016/j.cell.2013.04.037 23664763 PMC3709577

[17] Bernardo AS, Faial T, Gardner L, Niakan KK, Ortmann D, Senner CE, et al BRACHYURY and CDX2 mediate BMP-induced differentiation of human and mouse pluripotent stem cells into embryonic and extraembryonic lineages. Cell Stem Cell. 2011;9(2):144–55. 10.1016/j.stem.2011.06.015 21816365 PMC3567433

[18] Agarwal S, Holton KL, Lanza R. Efficient differentiation of functional hepatocytes from human embryonic stem cells. Stem Cells. 2008;26(5):1117–27. 10.1634/stemcells.2007-1102 18292207

[19] Lukovic D, Diez Lloret A, Stojkovic P, Rodriguez-Martinez D, Perez Arago MA, Rodriguez-Jimenez FJ, et al Highly Efficient Neural Conversion of Human Pluripotent Stem Cells in Adherent and Animal-Free Conditions. Stem Cells Transl Med. 2017;6(4):1217–26. 10.1002/sctm.16-0371 28213969 PMC5442830

[20] Noisa P, Raivio T, Cui W. Neural Progenitor Cells Derived from Human Embryonic Stem Cells as an Origin of Dopaminergic Neurons. Stem Cells Int. 2015;2015647437. 10.1155/2015/647437 PMC4430666 26064138

[21] Tao Y, Zhang SC. Neural Subtype Specification from Human Pluripotent Stem Cells. Cell Stem Cell. 2016;19(5):573–86. 10.1016/j.stem.2016.10.015 27814479 PMC5127287

[22] Cheng X, Ying L, Lu L, Galvao AM, Mills JA, Lin HC, et al Self-renewing endodermal progenitor lines generated from human pluripotent stem cells. Cell Stem Cell. 2012;10(4):371–84. 10.1016/j.stem.2012.02.024 22482503 PMC3580854

[23] Touboul T, Hannan NR, Corbineau S, Martinez A, Martinet C, Branchereau S, et al Generation of functional hepatocytes from human embryonic stem cells under chemically defined conditions that recapitulate liver development. Hepatology. 2010;51(5):1754–65. 10.1002/hep.23506 20301097

[24] Ang LT, Tan AKY, Autio MI, Goh SH, Choo SH, Lee KL, et al A Roadmap for Human Liver Differentiation from Pluripotent Stem Cells. Cell Rep. 2018;22(8):2190–205. 10.1016/j.celrep.2018.01.087 29466743 PMC5854481

[25] Chetty S, Pagliuca FW, Honore C, Kweudjeu A, Rezania A, Melton DA. A simple tool to improve pluripotent stem cell differentiation. Nat Methods. 2013;10(6):553–6. 10.1038/nmeth.2442 23584186 PMC3694177

[26] Guo G, Luc S, Marco E, Lin TW, Peng C, Kerenyi MA, et al Mapping cellular hierarchy by single-cell analysis of the cell surface repertoire. Cell Stem Cell. 2013;13(4):492–505. 10.1016/j.stem.2013.07.017 24035353 PMC3845089

[27] Moore FE, Garcia EG, Lobbardi R, Jain E, Tang Q, Moore JC, et al Single-cell transcriptional analysis of normal, aberrant, and malignant hematopoiesis in zebrafish. J Exp Med. 2016;213(6):979–92. 10.1084/jem.20152013 27139488 PMC4886368

[28] Norrman K, Strombeck A, Semb H, Stahlberg A. Distinct gene expression signatures in human embryonic stem cells differentiated towards definitive endoderm at single-cell level. Methods. 2013;59(1):59–70. 10.1016/j.ymeth.2012.03.030 22503774

[29] Levak-Svajger B, Svajger A. Investigation on the origin of the definitive endoderm in the rat embryo. J Embryol Exp Morphol. 1974;32(2):445–59. 4463213

[30] Gouti M, Tsakiridis A, Wymeersch FJ, Huang Y, Kleinjung J, Wilson V, et al In vitro generation of neuromesodermal progenitors reveals distinct roles for wnt signalling in the specification of spinal cord and paraxial mesoderm identity. PLoS Biol. 2014;12(8):e1001937 10.1371/journal.pbio.1001937 25157815 PMC4144800

[31] Turner DA, Hayward PC, Baillie-Johnson P, Rue P, Broome R, Faunes F, et al Wnt/beta-catenin and FGF signalling direct the specification and maintenance of a neuromesodermal axial progenitor in ensembles of mouse embryonic stem cells. Development. 2014;141(22):4243–53. 10.1242/dev.112979 25371361 PMC4302903

[32] Patani R, Compston A, Puddifoot CA, Wyllie DJ, Hardingham GE, Allen ND, et al Activin/Nodal inhibition alone accelerates highly efficient neural conversion from human embryonic stem cells and imposes a caudal positional identity. PLoS One. 2009;4(10):e7327 10.1371/journal.pone.0007327 19806200 PMC2752165

[33] Si-Tayeb K, Noto FK, Nagaoka M, Li J, Battle MA, Duris C, et al Highly efficient generation of human hepatocyte-like cells from induced pluripotent stem cells. Hepatology. 2010;51(1):297–305. 10.1002/hep.23354 19998274 PMC2946078

[34] Ring KL, Tong LM, Balestra ME, Javier R, Andrews-Zwilling Y, Li G, et al Direct reprogramming of mouse and human fibroblasts into multipotent neural stem cells with a single factor. Cell Stem Cell. 2012;11(1):100–9. 10.1016/j.stem.2012.05.018 22683203 PMC3399516

